# Syphilis in Pregnancy: The Reality in a Public Hospital

**DOI:** 10.1055/s-0038-1676569

**Published:** 2019-02

**Authors:** Rafael Garcia Torres, Ana Laura Neves Mendonça, Grazielle Cezarine Montes, Jacqueline Jácome Manzan, João Ulisses Ribeiro, Marina Carvalho Paschoini

**Affiliations:** 1 Department of Fetal Medicine, Universidade Federal do Triângulo Mineiro, Uberaba, MG, Brazil; 2 Department of Obstetrics and Gynecology, Universidade de Uberaba, Uberaba, MG, Brazil

**Keywords:** syphilis, pregnancy, congenital infection, sífilis, gestação, infecção congênita

## Abstract

**Objective:** The present study assessed epidemiological and obstetrical data from pregnant women with syphilis at the Hospital de Clínicas of the Universidade Federal do Triângulo Mineiro (UFTM, in the Portuguese acronym), describing this disease during pregnancy and its vertical transmission for future healthcare actions.

**Methods:** Records from pregnant women who had been admitted to the Obstetrics Department of the Hospital de Clínicas of the UFTM and were diagnosed with syphilis between 2007 and 2016 were reviewed. A standardized form was used to collect epidemiological, obstetric data and outcomes of congenital infection. The present research has been authorized by the Ethics Committee of the institution.

**Results:** There were 268 women diagnosed with syphilis, with an average age of 23.6 years old. The majority of the patients were from Uberaba. Inadequate prenatal care was observed in 37.9% of the pregnant women. Only 34.2% of the patients completed the treatment according to the guidelines issued by the Ministry of Health of Brazil, and 19.8% of the partners of the patients underwent adequate syphilis treatment; 37 (13.8%) couples (patients and partners) underwent correct treatment. Regarding the obstetric outcomes, 4 (1.5%) patients had a miscarriage and 8 (3.4%) had fetal losses (from the fetal loss group, 7 had no adequate treatment); 61 (25.9%) patients had premature births – this prematurity has been significantly correlated to inadequate or incomplete treatment in 49 (27.9%) patients, compared with 12 (13.0%) patients with premature births and adequate treatment (*p *= 0.006). The average live newborn weight was 2,840 g; 25.3% had a birth weight < 2,500 g; 74.2% had congenital syphilis, a data with heavy correlation to inadequate or incomplete prenatal care, prematurity, and low birth weight.

**Conclusion:** Public awareness policies on adequate prenatal care, intensification of serological screening, and early treatment of syphilis are needed, considering the rise of cases diagnosed during gestation and its potentially preventable deleterious consequences related to congenital transmission.

## Abstract (pt)

**Resumo:**

**Objetivo:** O presente estudo avaliou dados epidemiológicos e obstétricos de gestantes com sífilis no Hospital de Clínicas da Universidade Federal do Triângulo Mineiro (UFTM), objetivando o conhecimento desta infecção no ciclo gravídico e a transmissão vertical para futuras ações em saúde pública.

**Métodos:** Foram revisados registros de gestantes admitidas no Departamento de Ginecologia e Obstetrícia do Hospital de Clínicas da UFTM, diagnosticadas com sífilis entre 2007 e 2016. Para a coleta de dados, utilizou-se um formulário padronizado enfocando aspectos epidemiológicos, obstétricos e infecção congênita. A presente pesquisa foi autorizada pelo Comitê de Ética da instituição.

**Resultados:** Obteve-se 268 gestantes diagnosticadas com sífilis, com idade media de 23,6 anos, sendo a maioria de Uberaba. A assistência pré-natal foi inadequada em 37,9% dos casos. O tratamento para sífilis, de acordo com as diretrizes do Ministério da Saúde do Brasil, foi realizado por 34,2% das gestantes e por 19,8% dos parceiros. Quanto aos desfechos obstétricos, observou-se que 4 (1,5%) pacientes evoluíram com abortamento e 8 (3,4%) com óbito fetal, das quais 7 não realizaram tratamento. Observou-se parto prematuro em 61 (25,9%) gestantes, e a prematuridade foi significativamente associada ao tratamento ausente/incompleto, com 49 (27,9%) casos, comparada a 12 (13,0%) casos nos quais o tratamento foi adequado (*p *= 0,006). Quanto aos recém-nascidos, o peso médio foi de 2.840 g, e 25,3% apresentaram peso < 2.500 g. Diagnosticou-se infecção congênita em 74,2%, dos casos, associada significativamente ao pré-natal inadequado, ao tratamento ausente/incompleto, à prematuridade e ao baixo peso ao nascer.

**Conclusão:** Políticas públicas de conscientização sobre pré-natal adequado, intensificação de rastreamento sorológico e tratamento precoce da sífilis são necessárias, haja vista a ascensão dos casos diagnosticados na gestação e suas consequências deletérias potencialmente evitáveis relacionadas à transmissão congênita.

## Introduction

According to the World Health Organization (WHO), 1.5 million pregnant women are diagnosed with syphilis annually. Even though methods for laboratorial diagnosis and prenatal tracking guidelines are widely available and the treatment is relatively simple, congenital syphilis remains a global public health policy problem, with a significant newborn death rate.^1^ ^2^ Facts such as recent syphilis epidemics were reported around the world, including alarming increases in the infection rates in Brazil, meaning that improved care and the prevention of adverse effects are a substantial necessity.^3^ ^4^ ^5^

Syphilis, a sexually transmitted disease (STD) caused by the spirochete *Treponema pallidum*, goes through distinct phases from the moment of infection – primary, secondary, latent, and tertiary. During pregnancy, there is no alteration on the physical manifestations of any phase, and transmissibility is possible in any stage of the pregnancy.^6^ The risk of congenital syphilis depends on the concentration of the etiological agent in the maternal bloodstream, which is higher during the primary and secondary phases. Other factors, such as gestational age, maternal treatment, and fetal immunological response must be related to vertical transmission.^7^

Complications from syphilis may occur in the initial stages of pregnancy, such as miscarriages; intrauterine growth restriction, preterm labor, or fetal death are possible in subsequent phases.^6^ Syphilis must be thoroughly investigated in order to assess the infection and administer treatment, which can reduce fetal death rates when started early. During pregnancy, diagnoses are made in the same way as in the general population, using serological (treponemic or non-treponemic) tests.^8^ ^9^

Non-treponemic tests, such as venereal disease research laboratory (VDRL), rapid plasma reagin (RPR), unheated serum reagin (USR), and toluidine red unheated serum test (TRUST) use flocculation mechanisms in which antibodies against lipidic material from damaged cells and, possibly, from cardiolipin from treponemes are detected. These tests are more affordable and used for screening. However, they have low specificity and false positives due to damage to other cells are common.^9^

Treponemic tests, like the fluorescent treponemal antibody absorption test (FTA-ABS), detect immunoglobulin M (IgM) and immunoglobulin G (IgG) against *T. pallidum*, so syphilis can be identified sooner (∼ 3 weeks after contagion), and the results can remain positive even after adequate treatment. These tests are more specific for diagnosis than the non-treponemic ones but are not used for measuring therapeutic response.^10^ ^11^

Penicillin G benzathine is the first-line treatment. In allergies, desensitization is advised, and if the first-line treatment is not available, doxycycline and tetracycline are alternatives, but their usage is unadvisable in pregnant women.^7^ ^12^ Third-generation cephalosporin has been added as another treatment option.^13^

Serological screening and partner cooperation are extremely important in pregnant women diagnosed with syphilis, as to stop its further transmission. Screening for other STDs must be offered to the couple through serological tests and clinical examinations.^12^ There is no vaccine for syphilis, and previous infections do not give specific immunity.^9^ ^11^

In Brazil, 164,264 pregnant women have been diagnosed with syphilis from 2007 to 2016. In this period, the diagnoses had an ascending tendency, rising from 6,914 in 2007 to 33,381 in 2015. There were 104,351 congenital syphilis cases, a national average of 6.5 per every 1,000 live births, and 2015 had the highest incidence of congenital syphilis cases— (19,228).^4^ ^14^

During prenatal care, after the diagnosis of maternal syphilis, obstetric ultrasonography can be useful in order to identify suggestive, but unspecific signs of congenital infection. Those findings stem from an intense inflammatory response to *T. pallidum* and are only visible after the 20^th^ week of pregnancy, when the fetal immunological system is mature enough.^15^ ^16^ ^17^ About 31% of the infected fetuses may show these signs, and the most common are hepatomegaly (80%), fetal anemia (33%, evidenced by an increase in the fetal middle cerebral arterial peak systolic velocity in a Doppler assessment), and placentomegaly (27%). Polyhydramnios, ascites, and edema were also present in a smaller proportion.^18^ ^19^

The outcomes of congenital syphilis include non-immune fetal edema, jaundice, deafness, hepatosplenomegaly, rhinitis, cutaneous rash, and pseudoparalysis.^20^ Due to its high perinatal death rate, the WHO issued targets on the eradication of the vertical transmission of syphilis. Among the suggested actions were multidisciplinary interventions that could improve the access to healthcare to women, their partners and children, improvements in prenatal care, and screening for high-risk patients.^21^

## Methods

The present retrospective study assessed epidemiological and obstetrical data among pregnant women with syphilis in the Hospital de Clínicas of the Universidade Federal do Triângulo Mineiro (UFTM, in the Portuguese acronym), describing this disease during pregnancy and its vertical transmission for future healthcare actions.

Medical records from pregnant women diagnosed with syphilis in prenatal care and referenced to the Obstetric Infections Ambulatory of the UFTM or admitted to the Hospital de Clínicas of the UFTM between 2007 and 2016 were reviewed. The maternal data yielded epidemiological and obstetric information, syphilis infection profile, as well as treatment and pregnancy outcomes, while the fetal data collected information on postnatal infectious diagnosis. A structured and validated questionnaire, in which patients and newborns were anonymously identified through their medical record number, has been used to achieve the goals of the present research. The present study has been authorized by the Ethics Committee of the institution.

Patients with reactive FTA-ABS IgM or a reagent titer > 1:4 in the VDRL test were considered infected with syphilis. The definition for adequate prenatal care was the same used by the Ministry of Health of Brazil, which is > 6 appointments during pregnancy.

The treatment of the patient and/or of her partner was deemed adequate when it followed the guidelines of the Ministry of Health of Brazil, considering a weekly dose of 2,400,000 units of penicillin G benzathine during 3 weeks in inconclusive primary or secondary syphilis. After the treatment, there was a follow-up with VDRL analysis every 30 days.

Treatment during the prenatal follow-up was considered successful when the VDRL results showed two titers reduction in mass fraction after 3 months of treatment, or three titers reduction in mass fraction 6 months after the end of the treatment. Adequate treatment included the successful treatment of the partner of the patients and > 30 days between the end of the treatment and the birth. Data collected from the medical records of the newborns included gender, birth weight, and Apgar scores. Neonatal infection was defined by a reactive VDRL test from peripheral blood or liquor.

The dataset shown below underwent statistical analysis (chi-squared and Fischer exact tests).

## Results

**Fig. 1:**
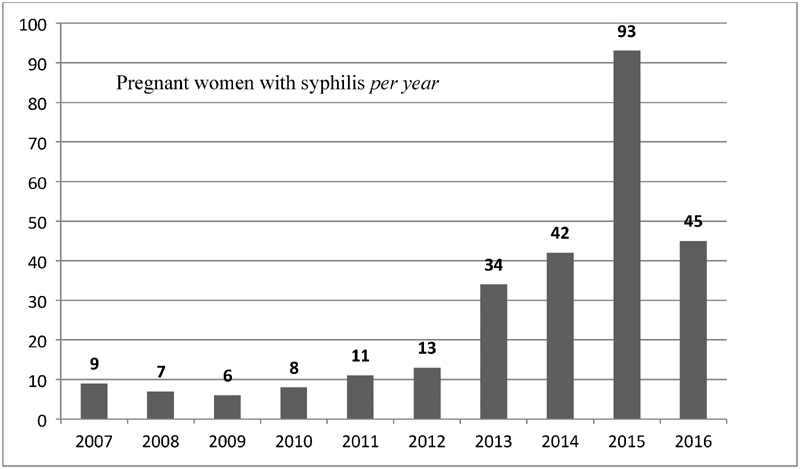
Distribution of pregnant women with syphilis between 2007 and 2016.

After the revision of the medical records, there were 268 pregnant women with syphilis from 2007 to 2016. Diagnoses were significantly higher in the last 3 years (2014–2016), with 178 (66.4%) diagnoses; 2015 had 93 (34.7%) of the diagnoses in the whole period, as shown in Fig. 1.

Regarding the epidemiological data, the median age of the patients was 23 years old, and the mean age was 23.6 years old. All of the patients were from Uberaba or its surrounding region (Triângulo Sul, in the state of Minas Gerais). Data from the Ministry of Health of Brazil on epidemiological vigilance in Uberaba shows that the patients were predominantly white (37.4%) and had incomplete middle schooling (23.3%).

Regarding the obstetrical data, from the 268 pregnant women included in the present study, 97 (36.2%) were in their first pregnancy, and 66 (24.6%) had > 4 pregnancies. Fifty patients (18.7%) reported previous miscarriages, and 78 (29.1%) had previous cesarean sections (CSs). Prenatal care was considered adequate in 180 (67.1%) patients.

The diagnosis of syphilis using non-treponemic tests (VDRL with a > 1:4 titer) and/or treponemic tests (FTA-ABS) had been made in the second trimester for a majority of patients (*n = *102; 38.1%); 86 (32.1%) patients were diagnosed in the third trimester. Out of the total sample, 98.2% of the patients had latent syphilis, as they showed no clinical signs or symptoms.

Using the criteria of the present research, 92 (34.3%) patients and 53 (19.8%) partners were considered to be adequately treated; only 37 (13.8%) couples (patients and partners) underwent correct treatment; the remaining patients informed they had no partner. It should be noticed that 176 patients (65.7%) had inadequate treatment or had not been treated at all during their prenatal screenings. Within the strata with adequate treatment, 11 (6.2%) used 1 g endovenous ceftriaxone daily for 10 days as an alternative treatment due to penicillin G benzathine shortages in Brazil.

Twenty-nine (10.8%) of all 268 patients gave birth in other hospitals, and 4 (1.5%) had a miscarriage. Of the 235 remaining patients, 173 (73.6%) had a timely (≥ 37 weeks) birth, and 61 (25.9%) had preterm births—of these, 36 (59.0%) had late preterm births (> 34 weeks, but < 37 weeks), and a single patient (0.4%) had post-term birth (in the 42^nd^ week of gestation). The median gestational age at birth was 38 weeks and 2 days. Regarding live births, 153 (65.1%) were vaginal births, and 82 (34.2%) were CSs. There were eight fetal deaths—three of them were inconclusive at necropsy, and the other one had general inflammations, villitis and pneumonia, suggesting an infection with maternal origin and placental transmission. The families of four stillborns did not allow the performance of an autopsy, but one obstetric ultrasonography within this group suggested hepatosplenomegaly.

Out of the 227 babies from syphilis patients, they were predominantly male (*n = *130; 58.8%); the average birth weight was 2,840 g (ranging from 2,832 g in the congenital syphilis stratum to 3,044 g in newborns without congenital syphilis), and 56 had low birth weight (< 2,500 g). Regarding the vitality of the newborns according to the Apgar index, 201 (91.0%) had a score > 7 on the first and fifth minutes; in the group with an Apgar score < 7, 17 (74.0%) were diagnosed with congenital syphilis. Regarding vertical transmission, data on 6 patients could not be assessed due to medical record digitalization issues; thus, out of the 221 live births, 164 (74.2%) had congenital syphilis

**Fig. 2:**
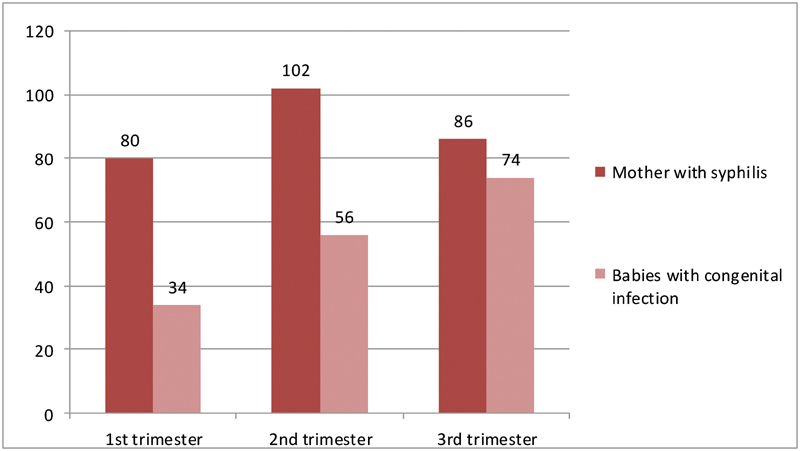
Mothers with syphilis and babies with congenital infection correlated to the time of diagnosis.

When the time of the maternal diagnosis was correlated with the neonatal infection, a trend towards a worsened prognosis (more infected babies) has been noticed in women diagnosed later in their pregnancies; 34 patients (42.5%) in the first trimester, 56 (54.9%) in the second trimester, and 74 (86.0%) in the third trimester had undesirable outcomes (Fig. 2). Among the 8 patients with fetal losses, maternal infection has been diagnosed predominantly on the second trimester (*n=* 5; 62.5%); 2 cases (25.0%) were diagnosed on the third trimester, and one (12.5%) case on the first trimester.

Regarding the treatment, when patients treated with ceftriaxone 1g daily for 10 days (*n = *11) were compared with the group treated adequately with penicillin G benzathine (*n = *92), the outcomes on congenital infection rates were similar: 45.6% in the first group, and 46.7% in the second group (*p *= 0.24). It should be noted that, in the group of 84 patients considered as receiving adequate prenatal care (≥ 6 appointments), there were 41 (48.8%) cases of congenital infection.

Prematurity (67 patients) had a significant correlation with inadequate treatment, that is, 49 (27.9%) cases, compared to 12 (13.0%) premature outcomes in patients who had adequate treatment (*p *= 0.006). Patients with adequate treatment (*n = *92) according to the criteria of the Ministry of Health of Brazil, had lower CS rates (23.9%) when compared with those that were not treated (35.1%; *p *= 0.062).

Regarding the outcomes of congenital infection, inadequate prenatal care, lacking or inadequate syphilis treatment, prematurity, and low birth weight were significantly associated with positive laboratorial diagnosis for syphilis in newborns, as shown in Table 1.

**Table 1:** Obstetrics and postnatal features related to the outcome of congenital infection

		Congenital infection		
		Present	Absent	*p-value*
Prenatal care	≥ 6 appointments	107	48	0.014
	< 6 appointments	56	10	
Treatment	Adequate	43	34	0.00001
	Lacking or inadequate	120	23	
Time of birth	< 37 weeks	47	7	0.010
	≥ 37 weeks	116	51	
Mode of birth	Cesarean section	62	20	0.558
	Vaginal	103	40	
Birth Weight	< 2,500g	51	5	0.0006
	≥ 2,500g	112	53	

## Discussion

From 2007 to 2016, there were 14,459 admittances to the Gynecology and Obstetrics unit of the teaching hospital of the UFTM (12,365 births and 2,224 curettages). There were 268 (1.9%) syphilis cases—21.7 for every 1,000 pregnancies. The Brazilian national average for the same period was of 6.5 cases per 1,000 pregnancies. These numbers are due to the status of the Hospital de Clínicas of the UFTM as a tertiary care regional center.

Epidemiological aspects of pregnant patients with syphilis show young women with lower education levels, mostly in their first pregnancy, and with a diagnosis of STD. Lack of STD preventive care shows up on their prenatal screenings—32.9% were deemed inadequate; this data is similar to the statistics of the Ministry of Health of Brazil from 2014, when 35.4% of the newborns were from mothers with inadequate prenatal care.^4^

In the present study, there was a statistically significant difference in congenital syphilis outcomes when adequate and inadequate prenatal screening groups were compared, even though the number of cases remained unacceptably high in both groups. It is not only the number of prenatal appointments, but also the timing in which they are done and the screening quality that play a decisive role in the control of vertical transmission.

A nationwide syphilis epidemic in pregnant women in the last few years has been reflected in the present study; there is an increase in the number of cases from 2013, when there was a 261% increase in comparison with the previous year. The year of 2015 had the higher incidence of cases (*n = *93), a number 10 times higher than the average from the years before 2013 and had 58.1 pregnant women with syphilis for every 1,000 live births, compared with the national rate of 11.2 per 1,000 births.^14^

The diagnosis of syphilis has been done mostly on the second trimester of gestation, followed by the third trimester. Nationwide statistics show that syphilis is mostly detected in the third trimester of gestation, reflecting the fact that the access to prenatal screenings and tests often comes later, showing late prenatal screenings and a lack of access to testing.^4^ There is a tendency towards inadequate treatment, as the time window between infection and treatment is larger and there is a therapeutic need to halt treatment 4 weeks prior to birth.

In addition to increases in the number of infected pregnant women, Brazil suffered shortages of the first-line treatment option for syphilis, penicillin G benzathine. Laboratories that make this medicine blame these problems on raw material shortages since 2014. Penicillin shortages are worrisome in a public health context of syphilis infections.

As an alternative therapeutic option, since doxycycline and tetracycline cannot be used during pregnancy, the Ministry of Health of Brazil issued a guideline indicating the use of ceftriaxone 1g daily for 10 days when penicillin is unavailable. Although longer and more expensive, this second-line treatment has been adopted by the Brazilian healthcare system due to supply shortages. Even when the treatment cycle is complete, the patient is still deemed to be inadequately treated as there were not, insofar, scientific subsidies to safeguard the efficacy of ceftriaxone 1g daily for 10 days as a treatment.

Endovenous ceftriaxone as a second-line treatment in 11 pregnant women had a similar prognosis to the first-line penicillin treatment on congenital infections. However, data remains insufficient on its efficacy due to the reduced number of cases when compared to patients treated with the first-line treatment.

According to the Ministry of Health of Brazil, the recommended treatment for pregnant women with probable latent or tertiary syphilis is at least three weekly doses of 2,400,000 UI penicillin G benzathine. Based on this, 92 (34.2%) pregnant women were deemed to be adequately treated, which is a low score, but still far superior to the dismal national average of 4.1% in 2015.

Treating partners of pregnant women with syphilis remains a challenge due to their low adherence to the treatment. The present study shows that only 53 (19.8%) partners at the moment of diagnosis underwent adequate treatment, which is above the national average (13.9%). Conjugal instability, absence of the partner in prenatal appointments, or reluctance to accept the treatment are constantly mentioned by patients as key factors for missing treatment. Thus, a high number of pregnant women were not deemed to be adequately treated—which makes the screening of newborns even more important.

Syphilis is generally linked to prematurity. Regarding pregnant women with syphilis, there was a rate of 25.9% preterm births, higher than the national average of 10.78% in 2015. Preterm births have been significantly higher in inadequately treated patients (27.9%; *p *= 0.006). Among the lethal consequences of syphilis, miscarriages and fetal deaths, mainly in the third trimester of pregnancy, were recorded in the present study. Even though the number of fetal autopsies has been low, in one case, the *causa mortis* has been directly linked to a transplacental transmission of a maternal infection of uncertain origin.

The predominant mode of birth was vaginal delivery, and there was no association between CSs and congenital infections. The average birth weight was adequate (2,840 g), and males were the majority; Apgar scores at the fifth minute from birth were favorable in 94.8% of the newborns.

In a study with 360 pregnant women with syphilis, regarding similar epidemiological and obstetrical factors, Qin et al^22^ show that, in the Chinese population, the average age of the pregnant women was higher (29.0 years old), and there has been a similar association between prenatal screenings, adequate treatment, and vertical transmission outcomes. However, the congenital infection rate was far smaller in the Chinese study (9.4%), when compared with a rate of 74.2% in the present study.^22^

Brazil, according to data from the Ministry of Health of Brazil, had a 6.5/1,000 infection rate from syphilis in newborns between 2007 and 2016. In the present study, the rates were 13.3 per 1,000 newborns, with higher rates in 2015 (34.3 per 1,000). In this same year (2015), in the United States, data from the Center for Disease Control and Prevention (CDC) show a 36.3% increase in congenital syphilis when compared with 2011, but with an incidence of 0.12 per 1,000 newborns, which is also lower than the Brazilian average.^4^ ^23^

The greater concentration of pregnant women with syphilis may arise from the fact that the Hospital de Clínicas of the UFTM is a reference center for high-risk pregnancy and maternal infections in the Brazilian national healthcare system (SUS, in the Portuguese acronym). The population served by the hospital includes Uberaba and 26 other cities from the region of the Triângulo Sul, with a total of 700,000 inhabitants, according to data from 2013 from the Health Regulation Department of Uberaba^24^. This fact must be noted when comparing local CS rates with the recommendations of the WHO, as there is a greater prevalence of comorbidities in the patients that may have to resort to a CS.

Among the limitations of the present study were the lack of socioeconomic and lifestyle data in the medical records of the women, and the lack of data regarding congenital infection when the birth had been conducted elsewhere. The quantity of data regarding the patients may have been overestimated because local healthcare services considered non-treponemic tests as conclusive diagnoses, which may yield false positives.

## Conclusion

The challenges for the Brazilian healthcare system are evident in this scenario, in which an alarming syphilis epidemic in pregnant women has taken place. Prematurity, fetal death and miscarriages – the most well-known consequences of syphilis – as well as the follow-up treatment for infected newborns, have potentially avoidable costs to the healthcare system and to the society. In spite of the investment of the government in awareness campaigns on prenatal care, the present study shows that the reality is still far away from ideal healthcare, as the access to adequate obstetrical assistance is still difficult. This scenario is troublesome in the context of STDs and may increase vertical transmission and heighten its consequences. The extremely low adherence to treatment among the patients and their partners is one of the main challenges to be overcome. Lower societal awareness regarding prevention and treatment shows an urgent need of educational policies that show a conjugal syphilis approach as crucial for preventing congenital infections. In spite of an apparent reduction in 2016, the situation is still alarming, given the high number of newborns affected over the last decade, and the constant threat of unavailability of the first-choice treatment, penicillin G benzathine. Since this epidemic must be controlled, ensuring the availability of treatments, as well as constant epidemiological surveillance and access to information, are decisive in order to control syphilis in Brazil.
